# Comparison of health-related quality of life measures in chronic obstructive pulmonary disease

**DOI:** 10.1186/1477-7525-9-26

**Published:** 2011-04-18

**Authors:** A Simon Pickard, Yoojung Yang, Todd A Lee

**Affiliations:** 1 Center for Pharmacoeconomic Research and Department of Pharmacy Practice, University of Illinois at Chicago, Chicago, IL, 60612, USA; 2 Center for Management of Complex Chronic Care, Hines Veterans Affairs Hospital, Hines, Illinois, 60141, USA

**Keywords:** respiratory disease, quality of life, COPD, health status, EQ-5D

## Abstract

**Background:**

The aims of this study were: (1) to compare the discriminative ability of a disease-specific instrument, the St. George's Respiratory Questionnaire (SGRQ) to generic instruments (i.e., EQ-5D and SF-36); and (2), to evaluate the strength of associations among clinical and health-related quality of life (HRQL) measures in chronic obstructive pulmonary disease (COPD).

**Methods:**

We analyzed data collected from 120 COPD patients in a Veterans Affairs hospital. Patients self-completed two generic HRQL measures (EQ-5D and SF-36) and the disease-specific SGRQ. The ability of the summary scores of these HRQL measures to discriminate COPD disease severity based on Global Obstructive Lung Disease (GOLD) stage was assessed using relative efficiency ratios (REs). Strength of correlation was used to further evaluate associations between clinical and HRQL measures.

**Results:**

Mean total scores for PCS-36, EQ-VAS and SGRQ were significantly lower for the more severe stages of COPD (*p *< 0.05). Using SGRQ total score as reference, the summary scores of the generic measures (PCS-36, MCS-36, EQ index, and EQ-VAS) all had REs of <1. SGRQ exhibited a stronger correlation with clinical measures than the generic summary scores. For instance, SGRQ was moderately correlated with FEV_1 _(*r *= 0.43), while generic summary scores had trivial levels of correlation with FEV_1 _(*r *< 0.2).

**Conclusions:**

The SGRQ demonstrated greater ability to discriminate among different levels of severity stages of COPD than generic measures of health, suggestive that SGRQ may provide COPD studies with greater statistical power than EQ-5D and SF-36 summary scores to capture meaningful differences in clinical severity.

## Background

Chronic obstructive pulmonary disease (COPD) is a leading cause of death worldwide and is associated with a high burden of illness [1], particularly in terms of health-related quality of life (HRQL). COPD is characterized by airflow obstruction that is not fully reversible and symptoms such as dyspnea, sputum production, and chronic cough [2]. Airflow limitation is usually progressive; thus daily activities can become very difficult as the condition gradually worsens. Consequently, the burden of COPD on HRQL disease tends to increase with COPD severity [3-6].

HRQL is inherently subjective, involving patient self-assessment of multiple dimensions of health that often are not strongly correlated with clinical indicators of COPD [7,8]. Measures of self-reported HRQL and pulmonary function assess different aspects of the disease and therefore provide complementary information [9,10]. Both generic and disease-specific HRQL instruments are used in COPD. St. George's Respiratory Questionnaire (SGRQ) is a disease-specific measure used in both COPD and asthma research [11]. EQ-5D [12] and the SF-36 [13] are generic measures of health often used in studies of COPD [3,5,10,14,15].

The severity of disease in a study population may affect the choice of instruments to measure health status. For instance, EQ-5D demonstrated fewer floor effects among patients with more severe asthma while SF-6D, a utility-based measure derived from items on the SF-36, demonstrated fewer ceiling effects and thus would be a more preferable measure to assess HRQL in patients with mild asthma who have good disease control [16]. A meta-analysis that examined EQ-5D index-based scores by COPD severity found that while mean scores decreased with the severity of GOLD stages, there was little discrimination of scores for moderate to severe stages of disease [15]. Such studies suggest that the performance of a HRQL measure may depend on the severity of COPD in a patient population. We were interested in further investigating the strengths and limitations of disease-specific and generic HRQL measures, particularly EQ-5D, SF-36 and SGRD, to better inform the selection of PRO measures in clinically heterogeneous COPD patient populations. Thus, aims of this study were: (1) to compare the discriminative ability of a disease-specific HRQL instrument (i.e., SGRQ) versus a generic instrument (i.e., EQ-5D); and (2), to evaluate the strength of associations among various clinical and HRQL measurements in COPD.

## Methods

### Subjects

We conducted a secondary data analysis of de-identified patient data from a study conducted in a Veterans Administration (VA) hospital. A previous publication described how the original data was obtained [17]. First, investigators identified patients with any inpatient or outpatient diagnosis of COPD in the previous 12 months and received VA care for at least 12 months prior to the study. Next, eligible patients were contacted by mail and received a follow-up phone call inviting them to participate in the study. If they consented, participants came to a pulmonary function laboratory where they completed pulmonary function testing, a 6-minute walk test (6MWT), and several self-reported measures, including the Borg dyspnea scale, SGRQ, SF-36, and EQ-5D. Respondents also completed a brief demographic questionnaire that asked about smoking history, including number of years that they smoked and average number of packs smoked per day. Number of pack-years was calculated based on number of years smoked (smoke-year) multiplied by average number of packs of cigarettes smoked per day (packs/day). As the present study was conducted using only de-identified data, it was granted exempt status by the UIC Office for the Protection of Research Subjects.

### Measures

We used forced expiratory volume in 1 second (FEV_1_) and forced expiratory vital capacity (FVC) to assess lung function. According to The Global Initiative for chronic Lung Disease (GOLD) guidelines [2], once airway obstruction is established based on a FEV_1_/FVC ratio of <0.70, COPD are categorized into 4 stages of disease: mild (FEV_1 _≧ 80%), moderate (FEV_1 _≧ 50-79%), severe (FEV_1 _≧ 30-49%), and very severe (FEV_1 _< 30%) [18].

FEV_1 _was expressed as a percentage of predicted normal values based on age, gender and height [19]. 6MWT is a widely used assessment of functional status in patients with COPD. It measures the distance (in meters) that a patient can walk on their own pace in six minutes. Dyspnea was measured on the Borg dyspnea scale [20]. Borg scores range from 0 (no breathlessness) to 10 (maximum breathlessness).

SGRQ is self-administered and includes 50 items in three components: symptoms, activity, and impact on daily life [21]. The SGRQ scores range from 0 to 100, with 0 indicating no impairment in the quality of life. Higher scores on the SGRQ represent worse HRQL. MID of four points was proposed for the SGRQ total score.

The veterans SF-36 is a slightly modified version of the SF-36 [13,22] that consists of 8 domains: general health, physical functioning, role function, role emotional, bodily pain, vitality, social functioning, and mental health. In addition, two summary scores, a physical component summary (PCS) and mental component summary (MCS) score can be calculated. The main modification made to the veterans SF-36 was to expand the number of response options from 2 to 5 for the role functioning scales due to physical health problems or emotional problems, which improved the properties of scales and the summary scores [23].

EQ-5D is a generic, preference-based utility instrument that includes a descriptor health classifier and a visual analog scale (VAS) [12]. The self-classifier has five dimensions including mobility, self-care, usual activities, pain/discomfort and anxiety/depression. An index-based utility score was calculated using algorithms for societal preference weights from the United Kingdom [24] and from the United States [25]. The VAS score is a rating of health today by the respondent where 0 represents worst imaginable health state and 100 represents best imaginable health.

### Statistical Analysis

Chi-square tests were used to test whether there were differences in patient characteristics for nominal variables across stages of COPD. Differences in means for continuous variables were examined using analysis of variance (ANOVA) and the non-parametric equivalent (Kruskal-Wallis) tests across the four stages of severity. Relative efficiency (RE) ratios were calculated by taking the ratio of the ANOVA-based test statistics, e.g. F-statistics, associated with the reference and comparator measure [26]. The SGRQ total summary score served as the reference measure in the calculation of RE ratios. Correlation between measures was calculated using Pearson's correlation coefficients (r). Strength of correlation was categorized as follows: absent (<0.20), poor (0.20-0.34), moderate (0.35-0.50) and strong (>0.50) [27]. A p-value < 0.05 was interpreted as statistically significant.

We hypothesized that the correlations between SGRQ and clinical measures, i.e. Borg dyspnea scale and 6MWT, would be stronger than between the summary scores of generic measures and the clinical measures, as SGRQ includes items specifically related to breathing problems. We also hypothesized moderate to strong correlations between the summary scores of the SGRQ, SF-36, and EQ-5D.

## Results

The mean (SD) age of the cohort was 71.3 (±10.3), and greater than 90% were white males. Patient characteristics did not differ across stages of COPD severity (Table 1). The exception was number of years smoked, which was significantly lower among patients with mildest stage of COPD (p = 0.02).

**Table 1:** Patient Characteristics

Characteristic Mean (SD)	Total Sample (n = 120)	GOLD Stage 1 (n = 23)	GOLD Stage 2 (n = 53)	GOLD Stage 3 (n = 27)	GOLD Stage 4 (n = 17)	p-value
Age	71.2 (10.3)	72.3 (11.5)	71.7 (11.4)	70.4 (8.4)	73.3 (7.9)	0.82^‡^
Male, n (%)	118 (98.3)	23 (100)	52 (98.1)	26 (96.3)	17 (100)	0.71^**†**^
Race, n (%)						0.18^**†**^
White	113 (94.2)	20 (87.0)	52 (98.1)	26 (96.3)	15 (88.2)	
Black	6 (5.0)	3 (13.0)	0 (0.0)	1 (3.7)	2 (11.8)	
Other	1 (0.8)	0 (0.0)	1 (1.9)	0 (0.0)	0 (0.0)	
Smoking status, n (%)						0.18^**†**^
Current	11 (9.2)	3 (13.0)	8 (15.1)	8 (29.6)	0 (0.0)	
Past	90 (75.0)	17 (73.9)	39 (73.6)	18 (66.7)	16 (94.1)	
Never	19 (15.8)	3 (13.0)	6 (11.3)	1 (3.7)	1 (5.9)	
BMI (Kg/m^2^)	29.1 (5.2)	30.6 (4.3)	29.6 (5.1)	29.2 (5.6)	25.3 (4.6)	0.01^‡^
Smoke-years (n = 109)	33.7 (15.0)	26.2 (13.0)	34.6 (15.2)	39.5 (13.3)	31.3 (16.1)	0.02^‡^
Packs/day (n = 105)	1.57 (0.83)	1.71 (1.10)	1.51 (0.88)	1.44 (0.60)	1.75 (0.68)	0.56^‡^
Pack-years (n = 109)	54.0 (37.9)	48.9 (46.0)	54.3 (42.2)	55.2 (26.2)	57.0 (32.7)	0.93^‡^

^†^based on Chi-square or ^‡^ANOVA

Mean FEV_1_, 6MWT, and Borg dyspnea scores were significantly different across GOLD stage (ANOVA/KWT, p-values < 0.001), with poorer functioning observed for patients with more severe COPD (Table 2). Mean symptom, activity, and impact and total SGRQ scores were significantly different across stages of disease, (ANOVA/KWT, all p-values < 0.001 except a p-value of 0.03 for symptom score), with activity and total scores getting worse with stage of disease. Mean SGRQ symptom and impact scores declined across stages 1 to 3, but stage 4 scores were slightly less severe than stage 3. Mean PCS and MCS scores both demonstrated a trend towards decline in health status with COPD severity, but only PCS mean scores were statistically different across COPD stage (p = 0.02). The mean EQ-5D index score (both UK and US) did not differ across the stages (p = 0.25). Mean EQ-5D VAS scores were different across stage of disease, with lower mean scores for more severe stage of disease (p = 0.02).

**Table 2:** Patient Clinical and Quality-of-Life Measurements (Total N = 120)

Measure mean (SD)	Total Sample (n = 120)	GOLD Stage 1 (n = 23)	GOLD Stage 2 (n = 53)	GOLD Stage 3 (n = 27)	GOLD Stage 4 (n = 17)	ANOVA		ANOVA	KWT
						
						F-stat	RE	p-value	p-value
FEV_1 _(%)	58.4 (24.8)	92.9 (13.7)	65.5 (9.1)	37.4 (5.7)	23.0 (4.8)	254.2		<0.001	<0.001
6MWT (m)	312.5 (108.0)	356.7 (124.3)	321.0 (95.8)	315.0 (98.8)	222.5 (89.6)	6.01		<0.001	<0.001
Borg Dyspnea	2.48 (1.64)	1.19 (1.11)	2.33 (1.65)	3.48 (1.67)	3.12 (0.49)	11.55		<0.001	<0.001
SGRQ Total	41.3 (19.7)	28.8 (15.0)	37.2 (18.6)	52.2 (19.6)	54.1 (13.5)	11.15	Ref	<0.001	<0.001
SGRQ Symptom	50.0 (24.1)	42.4 (21.4)	46.8 (23.9)	60.1 (26.1)	54.4 (20.2)	2.98	0.27	0.03	0.02
SGRQ Activity	57.3 (27.6)	38.0 (23.8)	53.5 (28.1)	65.7 (23.0)	82.5 (10.0)	12.36	1.11	<0.001	<0.001
SGRQ Impact	29.9 (18.9)	19.4 (14.9)	25.8 (16.3)	41.9 (20.0)	37.8 (17.5)	9.34	0.84	<0.001	<0.001
SF-36 PCS	34.4 (9.6)	39.5 (10.1)	34.4 (10.7)	32.4 (7.8)	30.9 (4.2)	3.49	0.31	0.02	0.002
SF-36 MCS	49.6 (10.9)	52.60 (9.4)	50.5 (10.4)	47.9 (12.5)	45.5 (10.7)	1.75	0.16	0.16	0.114
EQ-5D US Index	0.73 (0.19)	0.80 (0.13)	0.70 (0.21)	0.72 (0.19)	0.72 (0.16)	1.35	0.11	0.26	0.079
EQ-5D UK Index	0.63 (0.27)	0.73 (0.19)	0.59 (0.32)	0.63 (0.25)	0.63 (0.24)	1.38	0.12	0.25	0.069
EQ-5D VAS	65.3 (18.9)	74.3 (16.3)	66.2 (20.0)	60.1 (18.4)	58.7 (15.8)	3.31	0.30	0.02	0.004

GOLD: global burden of obstructive lung disease; Ref: Reference; RE: Relative efficiency ratio; SGRQ: St. George's Respiratory Questionnaire; ref: reference; ANOVA: analysis of variance; KWT: Kruskal-Wallis test

Using the SGRQ total score as the reference, relative efficiency ratios indicated that summary scores for SF-36 and EQ-5D were less efficient at discriminating between COPD stages (RE < 1) (Table 2). For the purpose of discriminating among COPD patients according to stage of disease, results indicated that only the SGRQ activity component score was more efficient than the SGRQ total score, i.e. RE > 1 (Table 2).

SGRQ activity and total scores demonstrated stronger correlations with the clinical measures than the other HRQL scores, although all HRQL measures had moderate to strong correlations with the dyspnea scale (Table 3). The summary scores of the generic measures - SF-36 PCS and MCS and EQ-5D index and VAS - were poorly correlated with FEV1 (r < 0.2), and poor-to-moderately correlated with 6MWT (r = 0.16 to 0.40). SGRQ total and impact scores were strongly correlated with both SF-36 and EQ-5D scores (r ≥ 0.5). The SGRQ symptom score exhibited moderate correlation with SF-36 and EQ-5D summary scores (0.35 ≤ r < 0.5). The correlation between the activity score and the generic instruments ranged from poor-to-moderate (0.2 ≤ r < 0.5).

**Table 3:** Correlations between Clinical and HRQL Measures

	**FEV**_**1**_	6MWT	Borg Dyspnea	SF-36 PCS	SF-36 MCS	EQ-5D index (UK)	EQ-5D index (US)	EQ-5D VAS	SGRQ Total	SGRQ Symptom	SGRQ Activity
FEV_1_	1										
6MWT	0.28^†^	1									
Borg Dyspnea	-0.41^§^	-0.21^†^	1								
SF-36 PCS	0.19^†^	0.40^§^	-0.58^§^	1							
SF-36 MCS	0.14	0.16	-0.34^§^	0.15	1						
EQ-5D Index(UK)	0.01	0.21^†^	-0.48^§^	0.51^§^	0.54^§^	1					
EQ-5D Index (US)	0.03	0.21^†^	-0.48^§^	0.51^§^	0.56^§^	0.99^§^	1				
EQ-5D VAS	0.16	0.31^§^	-0.48^§^	0.69^§^	0.49^§^	0.52^§^	0.53^§^	1			
SGRQ Total	-0.43^§^	-0.30^§^	0.76^§^	-0.67^§^	-0.50^§^	-0.55^§^	-0.57^§^	-0.60^§^	1		
SGRQ Symptom	-0.24^†^	-0.03	0.51^§^	-0.42^§^	-0.35^§^	-0.36^§^	-0.38^§^	-0.36^§^	0.73^§^	1	
SGRQ Activity	-0.45^§^	-0.46^§^	0.70^§^	-0.67^§^	-0.40^§^	-0.47^§^	-0.48^§^	-0.53^§^	0.88^§^	0.48^§^	1
SGRQ Impact	-0.36^§^	-0.21^†^	0.72^§^	-0.59^§^	-0.50^§^	-0.56^§^	-0.58^§^	-0.60^§^	0.94^§^	0.63^§^	0.72^§^

^† ^*p *< 0.05, ^§ ^*p *< 0.001

## Discussion

The results of this study supported the hypothesis that the disease-specific SGRQ had greater ability to discriminate among levels of COPD severity than generic measures of HRQL, i.e. SF-36 and EQ-5D. This finding is consistent with the other results that indicate the SGRQ is more strongly correlated with clinical measures than the summary scores of the generic measures. The correlation between generic HRQL summary scores - SF-36 and EQ-5D - and FEV_1 _was trivial, similar to a previous study [6]. GOLD stage is predicated upon breathing function, and the generic measures do not directly include items on breathing-related symptoms, while SGRQ does include such items. The RE ratios favoured the SGRQ total and activity scores, which suggests that those scales may provide greater statistical power to detect significant differences/changes in HRQL in COPD patients than the other measures, particularly if the study is intended to capture changes related to clinical severity.

Greater discriminative ability of disease-specific measures compared to generic HRQL measures has been reported in studies of other conditions [28-30]. In peripheral arterial disease, the disease-specific Vascular Quality of Life (VascuQol) measure was more discriminative than the EQ-5D and SF-36 [28]. In rheumatoid arthritis, Marra et al. found that the Rheumatoid Arthritis Quality of Life Questionnaire had greater ability to discriminate among the levels of severity of patients than the EQ-5D and SF-6D [30].

The EQ-5D VAS was better able to discriminate levels of HRQL according severity of disease than the EQ-5D index score in COPD patients. Unlike EQ-5D index-based scores, mean EQ-5D VAS scores decreased monotonically with stage of COPD, and the difference in VAS mean scores by severity represented what could be considered an important difference in VAS scores between stage 1, 2 and 3 [31]. It is important to note that COPD is often accompanied by other co-morbid conditions which were not captured in our data and may differentially affect the ability of HRQL measures to capture burden of illness.

Our study contributes to the literature on HRQL measurement in COPD in several ways. We present further evidence to support the validity of disease-specific and generic measures consistent with a previous study [5], but in a cohort of older and more severe COPD patients. Similar to Stahl and colleagues, we found that SGRQ total, PCS, and EQ-5D index and VAS scores got worse with severity based on GOLD stage [5]. Particular to this study, we showed that SGRQ scores were associated with greater statistical power to discriminate among levels of COPD severity using REs. We also found that the strengths of correlation between measures and EQ-5D index-based scores were nearly identical regardless of whether the UK or US value set was employed, because the correlation was nearly perfect (r = 0.993) between the EQ-5D index-based scores generated by each value set. For users of these measures, this study shows that the SGRQ has the advantage over generic measures in that it may be more likely to obtain a statistically significant result on a HRQL score if there are clinically meaningful differences/changes among patients. In addition, the EQ-5D index-based scores did not differentiate between the more severe stages of COPD. However, it is unclear if unobserved factors like comorbid conditions that might have been captured by the generic measure had a role in this finding.

This study had some limitations. The sample size used in our analyses may have yielded insufficient power to detect important differences across the severity stages. However, it was sufficiently powered to detect significant differences in EQ-5D scores [31]. Our data was cross-sectional; therefore, we could not compare the responsiveness of the measures over time. Use of a clinically-based measure of severity (GOLD stage) as the basis for comparing HRQL instruments may be suboptimal, but there is no clear gold standard for anchoring known-group comparisons of HRQL measures. Since the data used in our study were collected, modified versions of the SGRQ and EQ-5D have been introduced. These are all considerations for future studies comparing the psychometric performance of HRQL measures in studies of COPD patients.

## Conclusions

The SGRQ demonstrated greater ability to discriminate among different levels of severity stages of COPD and is more strongly correlated with clinical measures of COPD than generic measures of health. However, generic measures are intended to capture more broad aspects of health, and thus scores may potentially be less strongly correlated with clinical measures because they are capturing additional information on HRQL that is non-COPD related. For these reasons, generic and disease-specific measures may capture complementary information and it may be desirable to incorporate both types of measures in a study, depending on the goal of the study. As new versions of these widely used HRQL measures become available, such as a 5-level version of the EQ-5D, further comparisons - particularly using longitudinal data - will be useful in understanding the psychometric strengths and weaknesses of generic and disease-specific HRQL measures for the assessment and monitoring of COPD patient outcomes.

## Abbreviations

COPD: Chronic obstructive pulmonary disease; GOLD: Global burden of obstructive lung disease; HRQL: Health-related quality of life; FEV_1_: Forced expiratory volume in 1 second; FVC: Forced expiratory vital capacity; KWT: Kruskal Wallis test; RE: Relative efficiency; SGRQ: St. George's respiratory questionnaire; SF-36: Short-form 36 item questionnaire; VAS: Visual analog scale; 6MWT: six minute walk test.

## Competing interests

Simon Pickard is a member of the EuroQol group, a not for profit foundation that distributes the EQ-5D.

## Authors' contributions

ASP and TAL conceptualized the study, TAL obtained the data, ASP and YY analysed the data and drafted the manuscript. All authors provided input on the interpretation and they read and approved of the final draft of the manuscript.
